# *Cryptococcus*: A Rare Cause of Parotid Abscess in Liver Cirrhosis

**DOI:** 10.1155/2020/8849448

**Published:** 2020-11-17

**Authors:** Takeshi Okamoto, Katsuyuki Fukuda

**Affiliations:** Department of Gastroenterology, St. Luke's International Hospital, 9-1 Akashicho, Chuo-ku, Tokyo 104-8560, Japan

## Abstract

A 61-year-old man with decompensated liver cirrhosis presented with a parotid mass. Fine-needle aspiration of the parotid gland revealed *Cryptococcus neoformans*. Lumbar puncture confirmed cryptococcal meningitis. Multiple splenic lesions with radiographic features consistent with cryptococcal splenic abscesses were also seen. Despite antifungal therapy, the patient died 17 days after infection was confirmed. This is the first report of a cryptococcal parotid abscess in a patient with liver cirrhosis.

## 1. Introduction

*Cryptococcus* is an encapsulated yeast found worldwide. There are 2 *Cryptococcus* species which cause human diseases; *Cryptococcus neoformans* is responsible for over 95% of human cryptococcal diseases [1]. Infection is most often seen in human immunodeficiency virus (HIV) patients as the host response is primarily mediated by T cells. The pathogen is estimated to affect almost 1 million HIV patients every year [2]. Cryptococcal infection can also occur in other immunocompromised conditions such as liver cirrhosis as well as in immunocompetent people.

About 30% of cirrhotics die of infectious causes [3]. While *Cryptococcus* most commonly affects the lungs and the central nervous system, peritonitis is the most common presentation of cryptococcal infections in cirrhotics [4]. Reports of cryptococcal parotid abscess are extremely rare in both HIV and non-HIV populations. Here, we present the case of a cirrhotic patient with cryptococcal parotid abscess and meningitis, as well as multiple splenic lesions with radiographic features suggestive of cryptococcal splenic abscesses.

## 2. Case Report

A 61-year-old Japanese man presented to the emergency department complaining of abdominal pain and loss of appetite. His medical history was significant for decompensated liver cirrhosis (Child–Pugh C: 11 points) due to nonalcoholic steatohepatitis, gastric varices treated with balloon-occluded retrograde transvenous obliteration and percutaneous transhepatic obliteration, hypertension, and diabetes mellitus. Medications included diuretics, branched-chain amino acid supplements, ursodeoxycholic acid, and a proton pump inhibitor.

Upon presentation, he had a fever of 38°C and was mildly tachycardic. Physical examination was significant for a distended abdomen with no tenderness and edema of the lower extremities. Laboratory data basically remained unchanged from his baseline, remarkable for serum albumin of 2.6 g/dL, creatinine of 1.11 mg/dL, total bilirubin of 7.9 mg/dL, sodium of 129 mEq/L, hemoglobin of 10.3 g/dL, platelet count of 106,000/mm^3^, white blood count of 10,000/mm^3^, C-reactive protein of 2.2 mg/dL, international normalized ratio of 1.27, and ammonia of 47 *μ*mol/L. Serologic testing for HIV was negative. Paracentesis yielded clear ascites with minimal neutrophils. While cultures of blood and ascites (including fungi) were negative, intravenous antibiotics were given for suspected spontaneous bacterial peritonitis as no other cause of fever could be found. He was discharged 12 days later on oral antibiotics despite a persistent low-grade fever.

**Figure 1:**
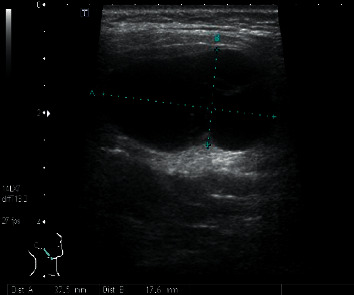
Ultrasonography of the right parotid gland revealed a 37.5 × 17.6 × 24.2 mm multilocular cystic lesion with clearly defined margins.

**Figure 2:**
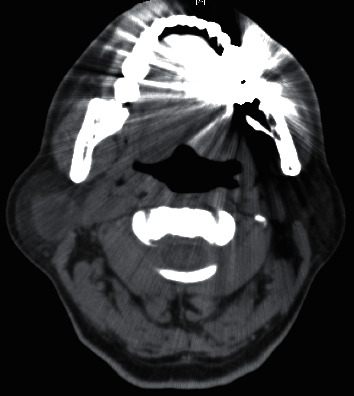
CT with contrast revealed an enhancing multilocular cystic lesion in the right parotid gland.

The patient was readmitted 8 days after discharge due to a high fever of 38.9°C. Weakness in the lower extremities and mild altered mental status were noted, initially believed to be due to hepatic encephalopathy, hyponatremia, or incipient sepsis. No meningeal signs were noted on physical examination. However, a new lump was observed in front of his right ear. The lump was soft and warm, but there was no tenderness. Ultrasonography of the right parotid gland revealed a 37.5 × 17.6 × 24.2 mm multilocular cystic lesion with clearly defined margins (Figure 1). Contrast CT of the neck revealed an enhancing multilocular cystic lesion in the right parotid gland (Figure 2). No masses, abscesses, or hydrocephalus were noted on contrast CT of the brain. Chest X-ray showed moderate pleural effusion limited to the right hemithorax. Fine-needle aspiration of the parotid lesion was performed, and 4 mL of yellow-green fluid was aspirated. Swelling and tenderness of the parotid gland resolved rapidly thereafter. Culture of the aspirate was positive for *Cryptococcus neoformans.*

**Figure 3:**
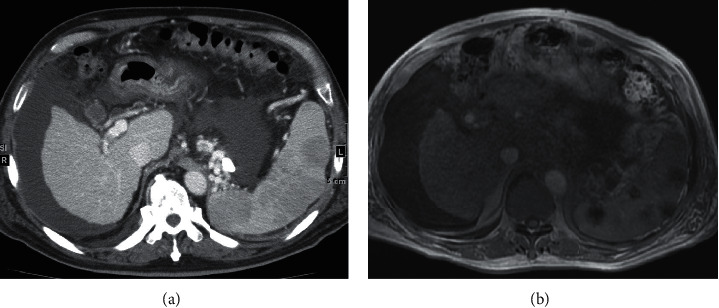
(a) CT with contrast revealed multiple small low-attenuation areas in the spleen. (b) MRI of the spleen revealed multiple low-intensity areas on T1-weighted imaging.

Lumbar puncture revealed xanthochromic cerebrospinal fluid (CSF) with an opening pressure of 22 cm H_2_O, a cell count of 100 cells/mm^3^ (39% neutrophils and 58% lymphocytes), pH of 8.0, protein of 109 mg/dl, and glucose of 20 mg/dl. Serum and CSF were both positive for the cryptococcal antigen, with a CSF titer of 1 : 8. India ink stain of CSF revealed an encapsulated yeast consistent with *Cryptococcus neoformans*. CSF culture was also positive for *Cryptococcus neoformans*. No growth was seen in blood, urine, and peritoneal fluid cultures. Upon detailed questioning, the patient stated that he worked in a fish market with frequent exposure to pigeons. CT with contrast showed multiple small low-attenuation areas in the spleen, suggestive of cryptococcal splenic abscesses (Figure 3(a)). Magnetic resonance imaging (MRI) of the spleen revealed multiple low-intensity areas more pronounced in the in-phase than in the opposed-phase on T1-weighted imaging (Figure 3(b)). A radiologist reviewed previous imaging studies and discovered multiple vague low-attenuation areas in the spleen on CT taken 4 months prior to admission. The patient was diagnosed with disseminated cryptococcosis.

The patient was started on liposomal amphotericin B and flucytosine. Lumbar puncture was repeated 11 days after commencing antifungal treatment, confirming negative CSF cultures for *Cryptococcus neoformans*. However, he died 23 days after admission and 17 days after the infection was confirmed. As repeated bacterial blood cultures remained negative and no other causes could be identified, disseminated cryptococcosis was determined to be the cause of death.

## 3. Discussion

Liver cirrhosis is known to suppress both innate and adaptive immune systems in many ways, known as cirrhosis-associated immune dysfunction syndrome [5]. Overactivity of regulatory T lymphocytes is known to arise in both compensated and decompensated cirrhosis, which may predispose the cirrhotic to bacterial and fungal infections [6]. Liver cirrhosis is thought to increase the risk of infection not only by depressing cell-mediated immunity but also by reducing phagocytosis, opsonization, and complement levels [7–9].

Retrospective studies suggest that 5–17% of non-HIV cryptococcal infections are associated with cirrhosis [7, 10]. Cryptococcal infection can arise regardless of etiology, including alcohol, hepatitis B, hepatitis C, and primary biliary cholangitis [11]. Other reported predisposing conditions include immunosuppressant use, malignancies, and diabetes mellitus, although one study suggested that diabetes was not a risk factor [7, 10]. As cryptococcal meningitis can be asymptomatic in as high as 43% of infected cases, a lumbar puncture should be conducted in all infected patients without contraindications [12]. Although a high index of suspicion is required, screening for cryptococcal antigenemia has been shown to be of little use, testing positive in none of 294 cirrhotics in a Korean study [13].

Risk factors in our case included decompensated liver cirrhosis, diabetes mellitus, and his chronic occupational exposure to pigeons. In one study, *Cryptococcus neoformans* was isolated in 34% of pigeon droppings on average and in 86% of pigeon droppings in some areas [14]. We had not suspected meningitis until cryptococcal infection was diagnosed due to the lack of meningeal signs and mild altered mental status which could easily be explained by other etiologies.

Cryptococcal infection in non-HIV patients is associated with a high mortality of about 27% despite recommended treatment with a formulation of amphotericin B combined with 5-flucytosine [12, 15]. Cryptococcal infections in liver cirrhosis carry a particularly poor prognosis. One report suggested a mortality rate of 81.2%, with most deaths directly attributable to cryptococcal infection and with over half of deaths occurring within 2 weeks after diagnosis [4]. Cryptococcal infection and abscess formation can affect almost any part of the body, with reports of bone, skin, intramuscular, cerebellar, brain stem, retropharyngeal, prostatic, ocular, breast, thyroid, ovarian, and mediastinal abscesses seen in the literature [16–25].

Cryptococcal parotid abscesses are very rare. A PubMed search revealed only 3 cases [26–28]. One involved an HIV patient, while the other 2 cases were immunocompetent. As in our case, all were noticed due to swelling on one side of the face, and all were diagnosed by fine-needle aspiration. None had a medical history of liver dysfunction or cirrhosis. The 2 immunocompetent cases recovered after antifungal therapy; the posttreatment course of the HIV case was not mentioned. While data are lacking, isolated parotid abscesses are easily drained percutaneously and may not affect prognosis.

A similar search revealed only 2 cases of cryptococcal splenic abscesses, possibly due to underdiagnosis [29, 30]. There may also be underreporting because of the difficulty in reaching a pathological diagnosis. Both involved HIV patients, of which one recovered with antifungals, and the other recovered after splenectomy but died 1 year later due to cryptococcal meningitis. Splenic fungal abscesses usually present as multiple small (less than 1 cm) low-attenuation areas on CT [31, 32]. In our case, the differential diagnosis included fungal abscesses, Gamna–Gandy bodies, and lymphoma. The low-attenuation areas were not observed on contrast CT taken 6 months earlier, suggesting rapid development. Each lesion was round with clear borders, which are not features of Gamna–Gandy bodies. Lymphoma may take various forms and could not be ruled out. While initially missed and not proven microscopically, splenic abscesses were the first visible manifestation of *Cryptococcus* in our case. The radiologic distribution may be explained by the fact that glucuronoxylomannan, the key component of *Cryptococcus neoformans* capsular polysaccharide, is initially stored in macrophages which subsequently localize in the red pulp of the spleen [33]. More attention to the spleen on imaging may lead to earlier diagnosis of disseminated cryptococcosis in some patients.

In conclusion, we report what we believe to be the first case of cryptococcal parotid abscess in a patient with liver cirrhosis. Identification of parotid swelling on physical examination led to prompt aspiration, culture, diagnosis, and subsequent treatment. A high index of suspicion is required to diagnose splenic cryptococcal abscesses. While exceedingly rare, these subtle pathologies may allow for quicker diagnosis and treatment of this lethal fungus.

## Data Availability

The data used to support the findings of this study are available from the corresponding author upon request.
